# Influence of acid rain and root reinforcement coupling factors on disintegration characteristics of expansive soil

**DOI:** 10.1371/journal.pone.0284269

**Published:** 2023-04-13

**Authors:** Yong-gang Huang, Gui-yao Wang, Shu Liu, Jian Tao

**Affiliations:** School of Civil Engineering, Changsha University of Science and Technology, Changsha, Hunan, China; REVA University, INDIA

## Abstract

The effect of soil fixation and anti-scour instability of slope vegetation generally depends on the strength and anti-disintegration ability of slope soil due to increase of root system. Therefore, it is particularly necessary to study the disintegration characteristics of expansive soil related to slope instability under acidic conditions (simulated acid rain). In this paper, the response surface method (RSM) was used with the pH value, root diameter, root length, root coefficient, and distribution as independent variables, and the disintegration amount of root-soil (DARS) after 60min as the response value. Then X-ray diffractometer (XRD) was used to analyze the mineral composition changes of the sample under this environment. Simultaneously, the plasticity index of expansive soil at different values of pH was studied to discuss the disintegration mechanism of root compound expansive soil in an acid environments. The results show that the root system improves the anti-disintegration characteristics of the root-soil, and the effects of various factors on the amount of disintegration were as follows: root length > pH value > root distribution > root amount > root diameter. The DARS with a length of 20mm increased by 26.67% and 41.56% compared to the 30mm and 40mm. Compared to the horizontal distribution and horizontal + slant distribution, the DARS with slant distribution was increases by 11.39% and 20.24% respectively. The DARS with 2 roots is increased by 9.92% and 16.75% compared to 4 and 6 roots respectively. The 1mm diameter DARS is 6.65% and 15.49% higher than the 2mm and 3mm, respectively. In addition, an acidic environments can lead to an increase in the amount of disintegration or rate of disintegration. The disintegration at pH = 4.2 was increased by 11.4% and 22.4% compared to pH = 5.6 and pH = 7, respectively. The acidity affects soil disintegration is due to the hydrophilic minerals in the expansive soil react with H^+^ ion in the acid solution to form soluble salts. Due to the dissociation and leaching of free quartz and metal oxides in the soil to varying degrees, the ability of expansive soil to accumulate is reduced. The intensity of erosion and leaching decreases with increasing pH. In addition, the pH value can affects the plasticity index of the soil, which increases with the increasing pH, thus affects the disintegration properties of the expansive soil.

## 1. Introduction

The area of acid rain area in China’ is about 530,000 square kilometers, according to the 2018 *China’s ecological environment bulletin*. It accounts for 5.5% of the country’s land area and includes 0.6% of the heavy acid rain area [1]. When acid rain penetrates into the soil, it can affect its properties. On one hand, the H^+^ ion in acid rain can leach calcium, magnesium, zinc, and additional elements combined with soil particles, making soil barren, in turn resulting in accelerated slope failure [2]. On the other hand, acid rain impact plays an crucial role in both detachment and transport of soil particles in soil erosion. It is well known that expansive soil and their engineering disasters have been a worldwide technical problem puzzling in the engineering geological field. The soil is more likely to be damaged than other soil slopes due to the characteristics of swelling and water loss shrinkage [3, 4]. Thus, ecological slope protection technology have receivd much attention in recent years. While the effect of soil fixation and anti-scour instability of slope vegetation commonly depends on the increase of root system on slope soil strength and anti-disintegration ability [5]. Therefore, it is particularly necessary to study the disintegration properties of expansive soil in acidic conditions, which are associated slope instability.

Rainfall has an effect on slope stability, but acid rain has a different effect on the mechanical properties of the soil than regular rainfall. In addition to the pore water pressure between the soil, it is also related to the mineral composition of the scoured soil and the structure of the soil [6–8]. For example, Chang et al. [9–11] analyzed the expansion and contraction characteristics, shear strength, and microscopic effect of expansive soil under dry-wet cycle under acidic conditions. It was concluded that the pH value of the acidic environment was negatively correlated with the expansion rate of expansive soil, but positively correlated with the shear strength. However, the amount of dry-wet cycles was positively correlated with the expansion rate at the early times, then stabilizes at the late times, with a large increas after two epochs of action. Bakhshipour et al. [12] studied the influence of acid rain intrusion on the compression performance of residual soil; Liu et al. [13–15] found the uniaxial compressive strength, elastic modulus, cohesion, internal friction angle, and triaxial compressive strength decreased with the increase of dry-wet cycles. Liu and Gui [16] concluded that the shear strength and cohesion of montmorillonite-quartz sand remolded soil decreased due to the reaction of H^+^ ion and montmorillonite under acidic conditions. Ivan and Ikuo [17] believed that acid pollution had a great influence on the strength characteristics of soil, and it had a significant influence on the stress-strain behavior of soil for the mineralogical composition of soil and the concentration of acid in pore fluid. Wei [18] analyzed the swelling and shrinkage deformation, shear strength parameters, stress-strain relationship curve, microstructure, and mineral element composition of remolded expansive soil under an acid environment. Then it was found that an acid environment could dissolve soluble salts and organic matter in expansive soil and alter its microstructure. And the stronger the acid environment was, the more the cohesion and internal friction angle decreased.

There have been a number of experimental studies on soil disintegration erosion. For example, disintegration [19–21] can aggravate slope erosion and potential erosion. Pore pressure and matrix suction [22] were the main controlling factors for the disintegration of unsaturated granite residual soil. Li et al. [23] established the correlation between effective void ratio and disintegration rate with the experimental research on the disintegration characteristics of highway slope loess during rainfall. Xiao et al. [24] explored the effects of initial water content, slope gradient, and effective root density of slope soil with mixed planting of forest and grass on the anti-disintegration ability of slope soil through the experimental study of the box root-soil model. Liu et al. [25] took four kinds of slope protection plants in loess plateau slope as the research object, then measured the vertical distribution of roots in the soil and mechanical indexes such as root tensile, the compression and disintegration resistance of undisturbed soil. Finally, it was concluded that the depth of soil and the root content in soil layer were the main factors affecting the rate of soil disintegration. Grassroots [5] have a significant role in enhancing the strength and anti-disintegration ability of root-soil complexes, and the effects of improvement are linearly related to root content. But the anti-disintegration ability of the remolded root-soil complex after structural damage is inferior than that of unperturbed soil. Xu et al. [26] found the modified nano-silicon materials have improved the unconfined compressive strength, shear strength, and anti-disintegration ability of loose sand. Zhu et al. [27] showed that the addition of dimer materials could improve the disintegration resistance of soil, and the disintegration resistance coefficient increased with the increase of material amount. Gu et al. [28] studied the effects of soil sample size, initial moisture content, water temperature, pH, and salinity on the disintegration characteristics of loess in the Heifangtai tableland. Zeng et al. [29] showed the comprehensive analysis about water content and temperature that water content is the main influencing factor of red clay disintegration in the natural climate range.

In summary, the acidic conditions can modify the microstructure and mineral composition of the soil, resulting in changes of strength and physical properties. These studies of the fundamental physical properties of remolded expansive soil show that the acidic environment has an effect on the mechanical properties and bulge properties of expansive soil. However, there have been few studies of the disintegration properties of expansive soil under acidic rain conditions, especially the study on the disintegration properties of expansive soil with vegetation roots. Root reinforcement can improve the anti-disintegration performance of soil, but an acidic environment can reduce its mechanical properties of soil. What is the effect of an acidic environment on the disintegration properties of root-soil? In recent years, ecological slope protection technology have received a lot of attention and have been tentatively applied to expansive soil slopes. Thus, this study is of engineering interest. In this paper, we have obtained the effects of solution pH, root diameter, root length, root amount, and root distribution on the root-soil disintegration properties. We used the expansive soil from Changsha, Hunan Province, as the research object, and simulated the acid rain environment in Hunan Province by preparing solutions with different pH. In addition, the XRD and liquid-plastic limit tests were carried out on expansive soil immersed in different values of pH to explore the mechanism by which acid affects the disintegration of expansive soil. The results can be used as a theoretical reference for expansive soil slopes in protective engineering.

## 2. Experimental method

### 2.1 Preparation of acidic solution

The type of acid rain in China is generally sulfuric acid [1]. Simultaneously, we collected the data of pH value of rainfall in central and eastern Hunan [30], thus the solubility of solution with 0.1 mol/L dilute sulfuric acid and 0.1 mol/L dilute nitric acid solution were mixed according to the volume ratio of 2:1. Then the pH of 3, 4, 4.2, 5, 5.6, and 6 were made respectively by adding distilled water to the mixed solution.

### 2.2 Sample preparation

The soil samples were taken from Changsha Shuxiang Road(112.979566,28.121945) Hunan, China. And its sample from 3 meters below the surface. It passes through a 2 mm sieve. The free expansion ratio, maximum dry density, liquid-limit water content, plastic-limit water content, and the optimum moisture content were determined according to the SL237-1999 Geotechnical Test Procedure. The results of test are shown in Table 1. According to JTJ031-95 "Classification of Highway Subgrade Design Code" (China), the soil can be classified as weak expansive soil. The sample is then prepared according to the optimal moisture content, maximum dry density, and 90% compaction degree. Due to the size effect of disintegration, the larger the size, the weaker the disintegration. However, the sample size is not clearly defined in the geotechnical test. Therefore the size of the disintegration sample was 61.8mm(diameter) * 20.0mm(height) for the convenience of sample preparation and highlight the role of roots.

**Table 1:** Basic physical parameters of the test soil.

Index	Optimum moisture content/%	Maximum dry density/(g·cm^-3^)	Liquid limit/%	Plasticity index	Free swelling ratio/%
value	20.5	1.61	53.5	29.6	48

The vetiver is used in roadbed slopes, reservoir slopes, and other protective structures due to its developed roots, fibrous roots, and strong growth ability [31]. Simultaneously, considering the characteristics of the high humid air temperature in the south, the vetiver roots were selected as adding roots to truly reflect the role of roots in the soil. Besides, the ring knife with 61.8mm(diameter) * 20.0mm(height) was used to take undisturbed soil at the depth of 2 m. It was wrapped in a preservative film to prevent water evaporation after the sampling was completed.

### 2.3 Test scheme

#### 2.3.1 The disintegration experiment of root reinforced soil under different pH value solution

The effects of different pH value (*X*_*1*_), root diameter (*X*_*2*_), root length (*X*_*3*_), root amount (*X*_*4*_), and distribution mode (*X*_*5*_) on the amount of disintegration (*R*) of expansive soil in solution at 60 min were obtained by the disintegration experiment of vanilla roots in expansive soil under acidic conditions. The value range of root length, diameter, and amount refer to the research results of Xiao et al. [24]. The level of each factor is shown in Table 2. It should be noted that the skew distribution is the connection between the top and bottom of the annulus, and the horizontal distribution is the horizontal midline position of the annulus. First, the production of soil sample is filled by two layers, with the root placed in the middle of the sample to simulate the horizontal distribution of roots. In making the tilting distribution, one-half of the expansive soil was taken, and the tilting plane was compacted with a geotechnical knife. Then, the roots were scraped, placed along the inclined plane, and then the expansive soil of the remaining was placed and compacted.

**Table 2:** Response surface analysis factor level table.

Coded value	pH value	Root diameter/mm	Root length/mm	Root amount	Root Distribution
-1	4.2	1	20	2	Slant
0	5.6	2	30	4	Horizontal
1	7.0	3	40	6	Horizontal + slant

**Fig 1:**
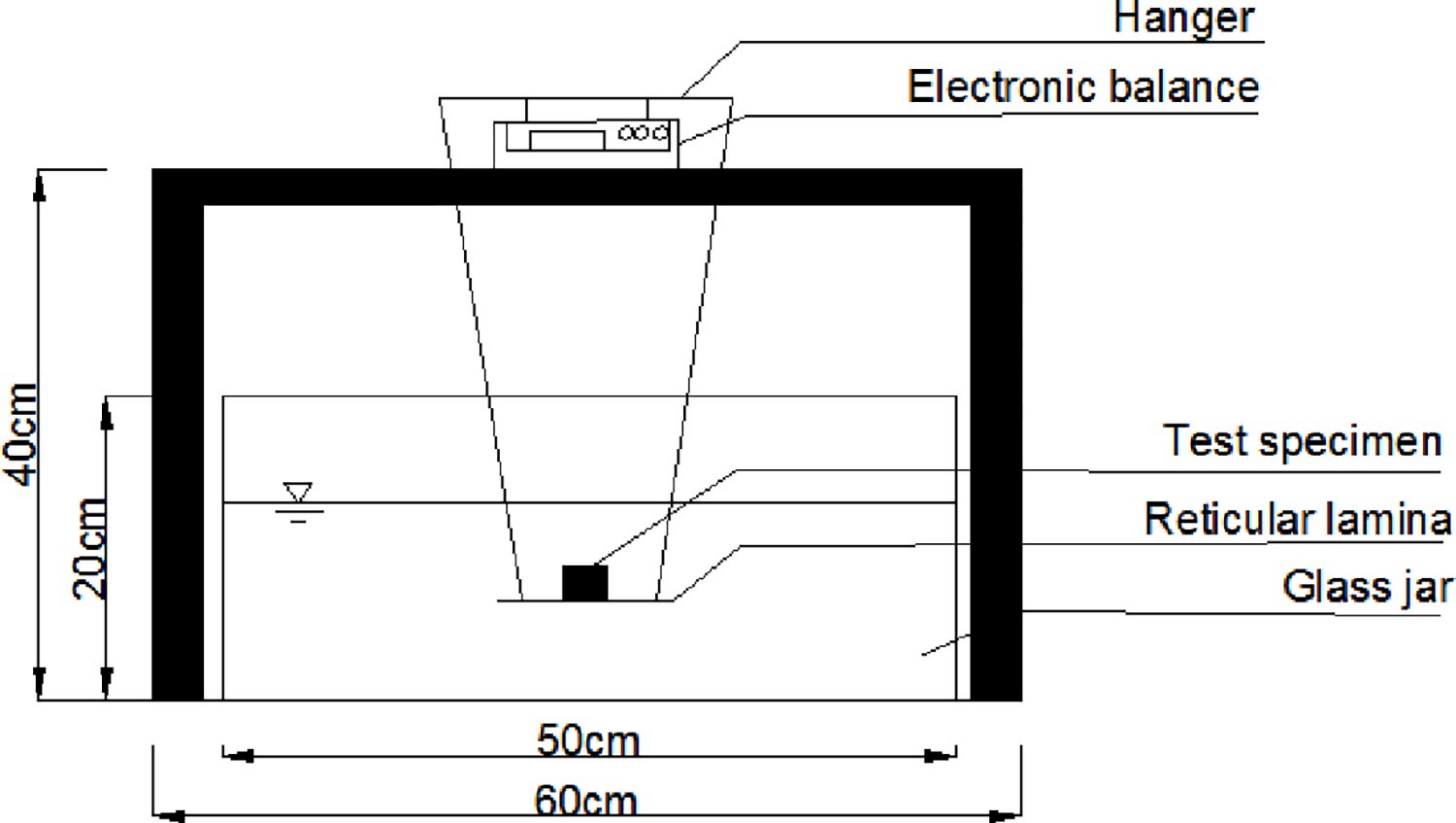
Schematic diagram of disintegration device.

The disintegration test setup is shown in Fig 1. An 8 mm grid plate was made with a diameter of 2 mm fine iron wire, which was connected to the hanger and the electronic balance by a fine wire. First, a readout of electronic balance were recorded at 0 min, and then the disintegration of the expansive soil was observed until 60 min. Readouts was recorded every 5 minutes for the first half hour, and for the second half hour. The amount of disintegration after 60 min was obtained by subtracting the remaining amount at 60 min from the reading after immersion in water. The test was repeated 6 times for each sample. Abnormal data are removed from the test data and the average value of the test results is taken. The test results are shown in Table 3.

**Table 3:** 60min disintegration test results.

No	pH value	Root diameter/mm	Root length/mm	Root amount	Root distribution	60min disintegration amount/g	No	pH value	Root diameter/mm	Root length/mm	Root amount	Root distribution	60min disintegration amount/g
1	5.6	2	30	4	0	57.4	22	5.6	2	30	6	1	46.1
2	7.0	2	30	4	-1	39.2	23	5.6	2	40	4	1	27.2
3	7.0	2	40	6	0	43.4	24	7.0	2	30	2	0	50.8
4	5.6	1	30	4	1	47.8	25	4.2	3	30	4	0	60.0
5	5.6	3	40	4	0	47.3	26	5.6	1	40	4	0	30.8
6	5.6	1	30	6	0	76.2	27	7.0	2	30	4	1	32.8
7	7.0	2	20	2	0	46.0	28	5.6	2	40	6	0	59.1
8	5.6	2	20	4	-1	78.7	29	4.2	1	30	4	0	66.0
9	5.6	1	20	4	0	79.4	30	5.6	2	40	4	-1	34.2
10	5.6	2	30	2	-1	76.0	31	7.0	2	30	6	0	38.8
11	5.6	2	20	6	0	62.8	32	5.6	2	20	4	1	79.8
12	5.6	3	30	2	0	49.4	33	7.0	1	30	4	0	51.0
13	4.2	2	30	4	-1	65.3	34	5.6	2	30	2	1	60.2
14	5.6	2	40	2	0	59.3	35	5.6	3	20	4	0	65.0
15	4.2	2	30	2	0	72.9	36	5.6	2	20	2	0	80.6
16	5.6	3	30	4	1	44.6	37	4.2	2	20	4	0	75.3
17	5.6	2	30	6	-1	44.8	38	4.2	2	40	4	0	41.0
18	4.2	2	30	6	0	61.4	39	5.6	3	30	6	0	44.3
19	5.6	1	30	2	0	61.4	40	5.6	3	30	4	-1	33.9
20	4.2	2	30	4	1	33.6	41	5.6	1	30	4	-1	40.0
21	7.0	3	30	4	0	42.4	42	7.0	-	-	-	-	100.8

The disintegrant fell to the bottom of the glass cylinder during the immersion disintegration test, and the balance reading decreased as the sample disintegrated. The reading (*m*_*t*_) is the remaining amount after the disintegration at a certain time(*t*).


$$m_{t}=m_{0}-m_{t}{}_{0}(1-\rho_{w}\frac{1+e}{d_{s}})+m_{wt}$$ (1)

Formula: *m*_*0*_ (g) is the instantaneous mass of sample immersion; *m*_*t0*_ (g) is the mass of the sample in the air after the disintegration time t; *d*_*s*_ is the Esample weight; e is pore ratio; *m*_*wt*_ (g) is the mass of the sample at time t.

#### 2.3.2 XRD analysis of expansive soil with different pH values

The undisturbed soil was immersed in an acidic solution with pH values of 3, 4, 5, and 6, respectively, and another solution of distilled water. It was soaked for 7 days. After 7 days, the samples were taken out for drying and milling before passing through a 0.075 mm sieve. Finally, soil samples were analyzed by XRD.

#### 2.3.3 Liquid plastic limit test of expansive soil at different pH values

First, the undisturbed soil samples were immersed in acidic solutions with pH values of 3, 5, and 6, respectively. Another solution was distilled water. The sample was taken out after being soaked for 7 days, and the liquid-plastic limit test was carried out according to the standard of the geotechnical test method (SL237-1999).

## 3 Results and analysis

It can be observed that the soil particles on the sample surface collapse when the test block invades into the disintegration solution, and the bubbles overflow on the sample surface. The surface bubbles increase significantly as the pH value of the disintegrating solution decreases. When the cracks appear in the middle of the disintegration, the amount of soil particles increases wand the bubbles on the surface of the sample decrease.

The disintegration process can be divided into three phases: an early slow disintegration stage, an intermediate rapid disintegration phases, and a late residual disintegration phases. The surface of the sample appears through cracks as in the successive disintegration experiment, and the sample is gradually divides into several small blocks connected by the roots of the plant. There is a mass increase process in the initial stage of the immersed disintegration test process. It is mainly due to that the water absorption rate of the non-saturated soil sample (water instead of air in the pore) is greater than its disintegration rate in the process of water absorption saturation. Then the rate of disintegration becomes larger than the rate of water absorption as the sample gradually saturates, and after a while disintegration no longer occurs.

Multiple regression fitting was performed on 42 groups of 60 min disintegration values (Table 3) by software, and the variance analysis table of block rate (Table 4) of the model was obtained. The P < 0.0001 of the model indicates that the model is significant and that different influence affect the 60 min disintegration amount. It can be seen that the effects of various factors on the amount of disintegration were as follows: root length> pH value> root distribution> root amount > root diameter(Table 4). The linear regression analysis of data(Table 3) was carried out, and the fitting function of 5 factors on the disintegration amount was obtained by Formula (2). The correction coefficient of the equation was 0.5494, indicating that the correlation of the model was reliable. Then the coefficient of variation is 22.42%, illustrating the high accuracy of the test. And the SNR is 6.6497 > 4, explaining that the equation can be a good reflection of the real experiments.


$$\begin{array}{l} R=403.73-31.21X_{1}-6.13X_{2}-83.33X_{3}-13.55X_{4}+9.79X_{5}-0.21X_{1}X_{2}+6.96X_{1}X_{3}-0.21X_{1}X_{4}-4.3X_{1}X_{5}+\\ \;3.8X_{2}X_{3}-2.48X_{2}X_{4}+0.35X_{2}X_{5}+4.35X_{3}X_{4}-2.05X_{3}X_{5}+4.3X_{4}X_{5} \end{array}$$ (2)

**Table 4:** Analysis of variance of 60min disintegration regression model.

Sources of variability	Sum of squares	Mean square	*F*	*P*
Model	5586.04	372.40	2.44	0.0184
*X*_*1*_	497.33	497.33	3.26	0.0812
*X*_*2*_	269.78	269.78	1.77	0.1939
*X*_*3*_	3207.49	3207.49	21.00	0.0001
*X*_*4*_	368.06	368.06	2.41	0.1311
*X*_*5*_	417.39	417.39	2.73	0.1087
*X*_*1*_ *X*_*2*_	0.9451	0.9451	0.0062	0.9378
*X*_*1*_ *X*_*3*_	256.68	256.68	1.68	0.2047
*X*_*1*_ *X*_*4*_	0.2368	0.2368	0.0016	0.9689
*X*_*1*_ *X*_*5*_	98.02	98.02	0.6417	0.4294
*X*_*2*_ *X*_*3*_	231.04	231.04	1.51	0.2283
*X*_*2*_ *X*_*4*_	98.01	98.01	0.6417	0.4294
*X*_*2*_ *X*_*5*_	1.96	1.96	0.0128	0.9106
*X*_*3*_ *X*_*4*_	75.69	75.69	0.4955	0.4869
*X*_*3*_ *X*_*5*_	16.81	16.81	0.1101	0.7424
*X*_*4*_ *X*_*5*_	73.96	73.96	0.4842	0.4919

**Fig 2:**
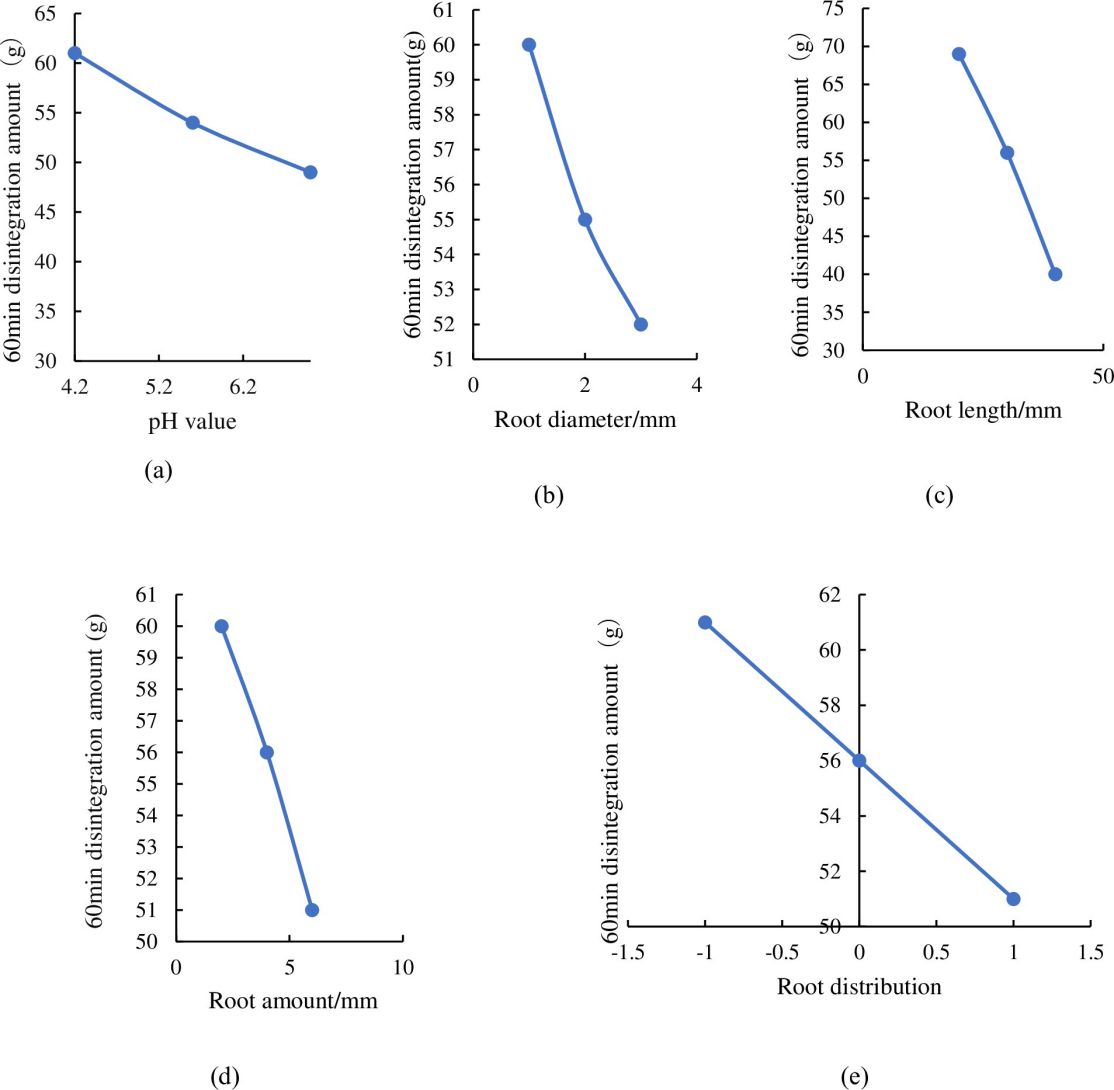
Single-factor analysis curve of 60min disintegration regression mode.

The soil completely disintegrated in pH = 7 solution at 22 min. In acid environment, the disintegration amount after 60 min was inversely proportional to the pH value, root diameter, root length, root amount, and distribution (Fig 2). It can be seen that the DARS in pH = 4.2 was 11.4% and 22.4% higher than that in pH = 5.6 and pH = 7, respectively (Fig 2A), indicating that acidic solution can significantly improve the ability of disintegration, and the stronger the acidity, the greater the quality of disintegration. It also can be seen that the DARS with a diameter of 1 was 6.65% and 15.49% higher than that of roots with a diameter of 2 and 3, respectively (Fig 2B), implying that the anti-disintegration ability of the root-soil complex increased accordingly with the root diameter increased. It show that the DARS with the length of 20 mm was 26.67% and 41.56% higher than 30 mm and 40 mm, respectively (Fig 2C), illustrating that the disintegration resistance of the root-soil complex was significantly improved with the root length increased. The DARS with 2 roots was 9.92% and 16.75% higher than that of 4 roots and 6 roots, respectively (Fig 2D), explaining that the anti-disintegration ability of the root-soil complex increased with the root amount increased. The DARS with slant distribution was 11.39% and 20.24% higher than that of horizontal distribution and horizontal + slant distribution, respectively (Fig 2E), indicating that the horizontal + slant distribution of root could improve the disintegration resistance of root-soil.

### 3.1 Mechanism analysis of acid rain affecting expansive soil disintegration

Firstly, raindrop impact (RI) that detaches and sheet flow that transports soil particles are the main processes causing soil erosion at the slope scales. RI can break the bonds holding the soil particles, and provide loose soil particles that can be transported by overland flow. In addition, raindrop impact influences the turbulence intensity and changes the detachment and transporting capacity of the sheet flow on a slope surface, which increases hillslope soil loss [32]. This is the direct behavior of acid rain. It is also part of the disintegration by the definition of disintegration. And this part of the disintegration is not the focus of this paper.

**Fig 3:**
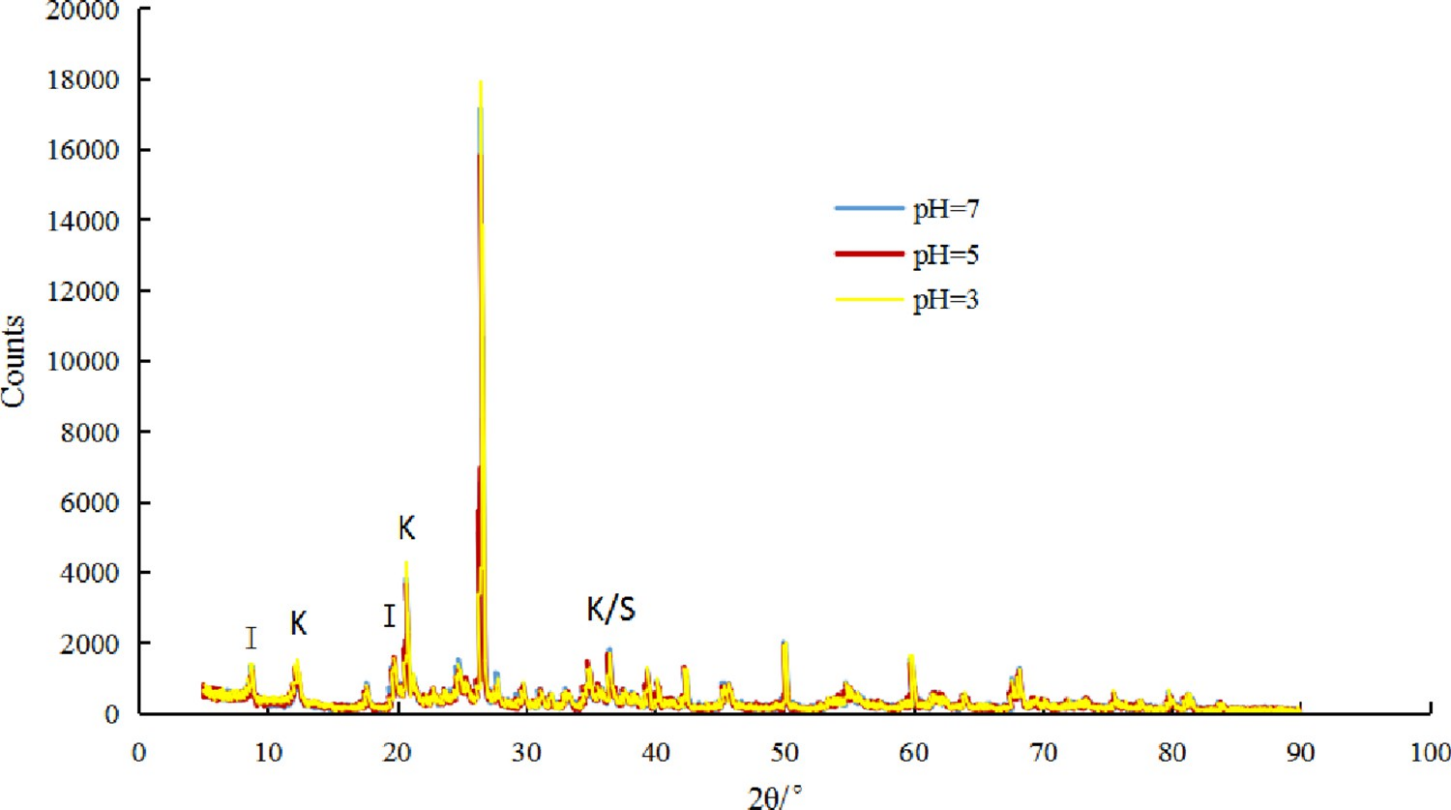
Comparison of XRD patterns of samples under acidic conditions; I is the illite, K is the kaolinite, and S is the quartz.

Secondly, the mechanism of acid rain affecting expansive soil is analyzed from the chemical mechanism. The diagram shows that this expansive soil were dominated by illite and kaolin (Fig 3). And the diffraction peaks of illite, kaolinite, and quartz mainly appear at 8°, 12°, 21°, 27°, and 34°, repetitively. The diffraction peak of mineral decrease with the decreasing of pH. Clay minerals like illite and kaolinite show a 33.1% and 29.3% reduction in diffraction peaks, respectively, and the quartz shows a 32% reduction in diffraction peak in acidic solution at pH = 3, compared with the solution at pH = 7.

Tests showed that the hydrophilic minerals in expansive soil react with H^+^ ion in an acidic solution to form soluble salts. In addition, free quartz and metal oxides in the soil are corrode and leach to varying degrees, resulting in a reduced stacking capacity of expansive soil. The corrosion and leaching effects are more obvious as the strength of the acidic solution increases. New pores and cracks were found in acid solution in expansive soil samples. The gaps between the soil particles become larger, and the pore water increases with the continued disintegration. And it eventually forms a penetrating crack that accelerates the disintegration of the sample. The lower the pH value of the solution, the greater the disintegration of expansive soil in the solution.

**Fig 4:**
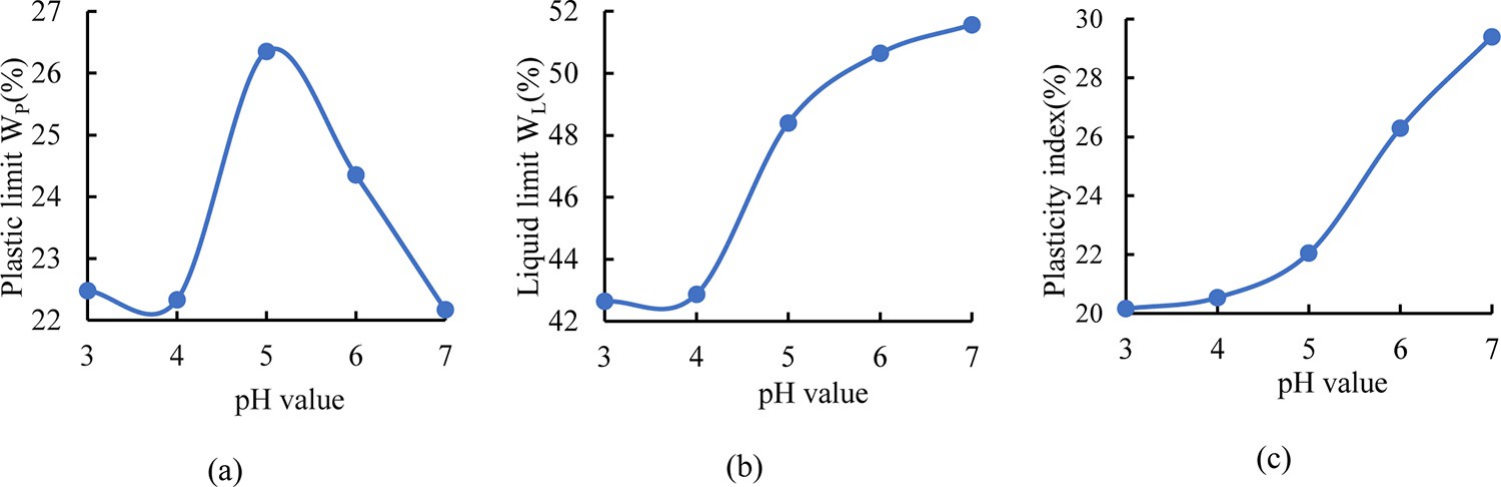
Liquid-plastic limit curves of expansive soil under different acidic environments.

Third, we analyze the mechanism of acid rain affecting the disintegration of expansive soils from the physical properties point of view. The plasticity of the sample increases firstly, then decreases with the increase of pH value, and reaches the peak at pH = 5 (Fig 4). The liquid limit and the plasticity index showed an ’S’ type increasing trend with the increase of pH value. The larger the plasticity index, the finer the particle. This indicates an increase in the specific surface area of the particles. If clay or hydrophilic mineral (such as montmorillonite) content is large, then the range of water content in plastic state is also large. Hence, the plasticity index can comprehensively reflect the effects of mineral composition and grain size of the soil. The disintegration velocity and rate of loss generally decreased with the clay content increased, indicating that pH affected the plasticity index of soil, thereby affecting the disintegration of the soil [33]. This study also confirmed that the greater the pH, the slower the disintegration of the expansive soil.

### 3.2 Influence mechanism analysis of vetiver root system on expansive soil disintegration

It was observed that the infiltration water bypassed the pores or larger pore channels surrounded by solid particles [34]. The soil absorbs water to forms a film water in a moist state. Moreover, the increased penetration time leads to the thickening of the water film. The pore channel is truncated and then extended to both ends until the pore channel is filled, and the residual air is compressed in the pore or wider pore. Lu et al. [35] put forward similar views on infiltration research. In conclusion, the main cause of soil disintegration is that the water content of the percolating soil particles reaches a wet state from the air-dry state, which leads to an increase of pore pressure enclosed in the soil.

The disintegration test of grass roots on soil [5] shows that the influence of vegetation roots on the disintegration effect is mainly reflected in two aspects: the root can be used as a water passage and the root wrapping effect on soil. When the soil reaches a moist state, the percolating water forms a film of water on the surface of the soil particles. It also enters the macropore through the root system at the same time. Compared with bare soil, grass root promoted infiltration [36]. When the water film-thickening pore channels are cut off, the macromolecules already have access to large amounts of water through the root system. As a result, the pore water pressure of the vegetative root-strengthened soil is smaller than that of the bare soil, which plays a role in enhancing the soil’s anti-scour effect. When the pressure of the formed pore water is greater than the binding force between the soil particles during the rainwater infiltration process, it causes the soil to disintegrate. In addition, the strengthening effect of the cross-root increases the binding force between the reinforced soil particles, thus inhibiting soil disintegration.

Gai et al. [37] studied the influence of different root-embedding methods on the shear strength of composite soil. It has also been shown that the study showed that the shear strength of the composite soil under the composite distribution is higher than that of the vertical and horizontal distribution. Therefore, it can be considered that the anti-disintegration capacity of the composite soil under the composite distribution is greater than that under the slant and horizontal distribution. But it should be noted that in practical engineering, the root system not only enhances the shear strength of soil but also increases the matric suction of soil [38]. The actual anti-disintegration capability is greater than that of the laboratory test. The root content was beneficial to improve the shear strength of soil, which well explained that could improve the anti-disintegration ability of expansive soil to a certain extent with the increase of length, diameter, and amount.

It should be considered that acid rain not only damages the structure of expansive soil (Figs 3 and 4) but also affects the growth of plants. Acid rain [39] made the chlorophyll content of vetiver decrease significantly. At pH = 3.5, this is the critical point that affects the photosynthetic rate, transpiration rate, and water efficiency of the vetiver. When the pH value > 3.5, which had no obvious effect on plants, but when it < 3.5, it had a significant effect on the plant. And it promoted photosynthetic rate when the pH > 4.5 [40]. Vegetation can enhance the disintegration of expansive soil. And the greater the pH is, the better effects for plants and expansive soil (Fig 4). Therefore, it is feasible to adopt vegetation slope protection for weak expansive soil slopes in areas with weak acidity.

## 4. Conclusion

The effects of acidic environment and root parameters on the disintegration properties of root-reinforced expansive soil have been studied through a laboratory disintegration tests. The nature of this phenomenon was discussed through XRD analysis and liquid-plastic limit tests. The following conclusions are finally obtained.

1. The response surface model developed in this paper takes into account the effect of solution pH values and root parameters on DARS in a comprehensive way. Even the prediction accuracy of the model is high and can reflect the real test conditions.
2. The root system improves the anti-disintegration characteristics of the root-soil, and the effects of various factors on the disintegration amount were as follows: root length> pH value> root distribution> root amount> root diameter.
3. Acidic environments can promote root-soil disintegration. For pH = 4.2, however, the amount of disintegration was 11.4% and 22.4% higher than for pH = 5.6 and pH = 7, respectively.
4. The effects of dissolution and leaching are more pronounced as the strength of the acid solution increases. The diffraction peaks of clay minerals such as ilmenite and kaolinite decreased by 33.1% and 29.3%, respectively. In contrast to pH = 7, quartz is reduced by 32 per cent at pH = 3.
5. The value of pH affects the plasticity index of the soil and thus the soil disintegration.

## Supporting information

S1 Data (XLS)
